# Atomic Force Microscopy: A New Look at Pathogens

**DOI:** 10.1371/journal.ppat.1003516

**Published:** 2013-09-05

**Authors:** David Alsteens, Audrey Beaussart, Sofiane El-Kirat-Chatel, Ruby May A. Sullan, Yves F. Dufrêne

**Affiliations:** Université catholique de Louvain, Institute of Life Sciences, Croix du Sud, 1, Louvain-la-Neuve, Belgium; Duke University Medical Center, United States of America

## Introduction

Microbial cells are highly complex and heterogeneous systems. In general, cell populations contain subgroups of cells which exhibit differences in growth rate as well as resistance to stress and drug treatment [1]. In addition, individual cells are spatially organized and heterogeneous, and this cellular heterogeneity is used to perform key functions [2]. This complexity emphasizes the need for single-cell analysis techniques in microbial research. Fluorescence imaging is a powerful tool to localize molecules in single cells [3], [4], but the resolution remains limited to the wavelength of the light source. On the other hand, high-resolution images of microbial structures can be obtained by electron microscopy techniques. In particular, cryo-electron tomography—or three-dimensional (3-D) electron microscopy—provides images of whole bacterial cells, at resolutions that are one to two orders of magnitude higher than those obtained with light microscopy [5].

In the past 20 years a new form of microscopy, atomic force microscopy (AFM), has revolutionized the way researchers probe the microbial cell surface. Instead of using an incident beam, AFM measures the minute forces acting between a sharp tip and the sample [6]–[8]. To generate a topographic image, the tip is attached to a cantilever that bends under force and is moved in three-dimensions using a piezoelectric scanner. While scanning the sample surface, cantilever's bending is measured by a laser beam focused on the free end of the cantilever and reflected into a photodiode. Unlike other microscopy techniques, 3-D images of cells and membranes are obtained at high resolution without staining, labelling or fixation, thus in physiological conditions.

AFM is much more than a surface-imaging tool in that it also measures the localization and mechanical properties of the individual cell surface molecules. In this modality, known as single-molecule force spectroscopy, the cantilever deflection is recorded as a function of the vertical displacement of the scanner (as the sample is pushed towards the tip and it retracts) [6], [7]. This results in a cantilever deflection vs. scanner displacement curve, which is transformed into a force-distance curve using appropriate corrections. The characteristic adhesion force between tip and sample measured during retraction is used to probe the distribution and mechanics of single molecules, such as cell surface receptors. These novel AFM techniques complement traditional methods used to analyse microbial cell walls and provide new opportunities for understanding cell surface interactions. In this review, we provide a flavour of the various applications offered by AFM in microbiology, and we highlight some of the key breakthroughs the technique has enabled in pathogen research.

## Imaging the Cell Surface

There has been rapid progress in recent years using AFM for imaging microbial surfaces under physiological conditions. In structural biology, AFM has provided fascinating insights into the supramolecular architecture of membrane proteins, thereby complementing data obtained by electron and X-ray crystallography [6]. In cellular microbiology, the technique has enabled researchers to unravel the surface structure of a variety of microbial species [7], [8]. A critical prerequisite for live cell imaging is sample preparation. The cells must be well attached to a suitable solid substrate, which can be achieved by mechanically trapping the cells into the pores of a porous polymer membrane. Using this method, AFM images were obtained for single cells from *Lactobacillus rhamnosus* GG, *Lactococcus lactis*, and *Lactobacillus plantarum* [9]–[12]. These three Gram-positive bacterial species exhibited major differences in cell surface architecture, reflecting differences in cell wall composition. *L. rhamnosus* GG had a rough surface morphology decorated with 15 nanometer–high wave-like structures, attributed to the production of extracellular polysaccharides involved in host adhesion [9]. By contrast, *L. lactis* showed 25 nanometer–wide striations documenting peptidoglycan cables running parallel to the short cell axis, thus supporting the classical model for peptidoglycan organization [10]. Similar nanocables were observed on purified sacculi from *Bacillus subtilis* [11]. Images obtained for *L. plantarum* documented a highly polarized surface morphology (smooth poles vs rough side walls) that was correlated with a heterogeneous distribution of teichoic acids [12].

Remarkably, high-resolution imaging has allowed researchers to observe cell surface structures to a resolution of a few nanometers. Observed structures include *Bacillus thuringiensis* flagella involved in cell motility [13], clustered proteinaceous microfibrils (“rodlets”) on the surface of the pathogen *Aspergillus fumigatus* [14], and hexagonal S-layers on *Corynebacterium glutamicum* [15]. As AFM images are directly obtained in buffer, acquiring sequential images of the same cell can reveal cell surface dynamics during growth. For instance, the surface of *Staphylococcus aureus* was observed during growth, revealing ring-like and honeycomb structures at 20 nanometers resolution [16], [17]. In another elegant work, AFM images of *Bacillus atrophaeus* spores revealed germination-induced alterations in spore coat architecture and disassembly of rodlet structures [18]. An exciting approach in current microbial research is to combine AFM with fluorescence microscopy. Such correlated AFM-fluorescence imaging was recently used to capture the different stages of interaction between *Candida albicans* and macrophages [19], including initial intercellular contact, internalization of yeast cells, intracellular hyphal growth, and mechanical piercing of the macrophage membrane resulting in pathogen escape. AFM disclosed nanoscale structural features of the macrophage surface that were not visible in the optical images, including ruffles, lamellipodia, filopodia, membrane remnants, and phagocytic cups.

AFM imaging thus opens up unprecedented possibilities for studying the structural details of the surface of microbial pathogens without drying, staining, or fixation. As conventional AFM imaging is limited by a rather poor temporal resolution, an important challenge is to increase the speed of AFM to study fast dynamic biological processes [20]–[22]. In microbiology, fast AFM instruments have recently enabled the observation of dynamic molecular processes in photoactivated bacteriorhodopsin [20] and magnetotactic bacteria [21], and the visualization of the initial stages of the action of antimicrobial peptides on *Escherichia coli* cells [22].

## Localizing Cell Surface Receptors

While AFM imaging lacks biochemical specificity, single-molecule force spectroscopy with functionalized AFM tips makes it possible to map the distribution of specific molecules on living cells. The method involves scanning the cell surface with a tip bearing cognate ligands while measuring specific receptor-ligand forces [7]. These single-molecule experiments have provided new insights into how cell surface adhesins are spatially organized and how this organization is used to control key functions like cell adhesion. Force spectroscopy with tips functionalized with specific antibodies was used to unravel the distribution of outer membrane cytochromes on *Shewanella oneidensis* bacteria [23]. In the context of tuberculosis research, this method enabled researchers to map the distribution of mycobacterial adhesins engaged in host interactions [24]. The adhesins were concentrated into nanodomains, presumably promoting the recruitment of receptors in host cells. Probing single Als adhesins from *C. albicans* with AFM tips terminated with specific antibodies triggered the formation and propagation of adhesion nanodomains on the cell surface [25]. This suggests that force-induced clustering of adhesins could be a general mechanism for activating cell adhesion in microbial pathogens. These studies show that single-molecule AFM has great potential for studying the distribution of microbial cell surface receptors and their dynamic remodelling in response to stress, such as drug treatment and mechanical force.

## Measuring the Mechanical Properties of Single Adhesins

**Figure 1:**
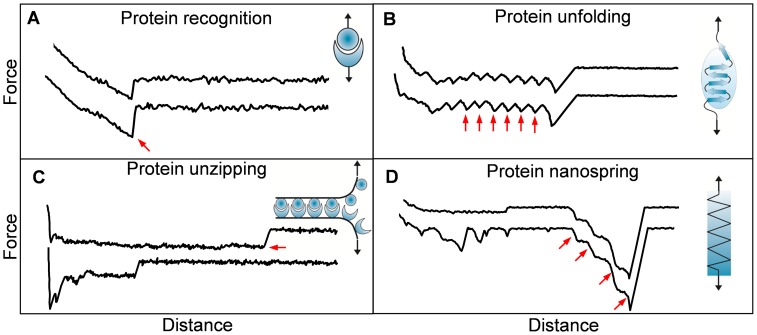
Single-molecule force spectroscopy experiments unravel the nanomechanics of microbial adhesins. Series of force-distance profiles obtained by stretching adhesins from various microbial species (see text for details): single adhesion peaks reflecting specific recognition (A), sawtooth patterns with multiple force peaks corresponding to the force-induced unfolding of protein secondary structures (B), constant force plateaus originating from the mechanical unzipping of amyloid interactions formed between multiple adhesins (C), and single large adhesion force peaks with linear shapes obtained by pulling on Gram-positive bacterial pili (D). The arrows emphasize the characteristic force peaks in each case.

The mechanical properties of proteins play essential roles in cellular interactions [26], [27]. Well-known examples are receptor-ligand bonds (“catch bonds”) from *E. coli* fimbrial adhesive protein FimH which strengthen under external mechanical force [27]. To date, the molecular mechanisms by which pathogens use cell surface proteins to sense and respond to physical cues are still poorly understood. With its ability to stretch cell surface proteins, single-molecule force spectroscopy has recently contributed to address this issue. Microbial adhesins of different species show very different mechanical signatures, including specific recognition, unfolding, unzipping, and nanospring-like behaviour (Figure 1), further described hereafter. Force spectroscopy experiments between mycobacterial adhesins and heparin receptors yielded single adhesion peaks reflecting the rupture of specific adhesin-receptor bonds (Figure 1A) [24]. FimH adhesins were shown to bind to mannosylated surfaces via catch bonds, through a mechanism in which force induces an allosteric switch to the high-affinity binding conformation of the proteins [28]. Stretching single Als adhesins from *C. albicans* revealed sawtooth patterns with multiple force peaks, corresponding to the force-induced unfolding of hydrophobic tandem repeats engaged in cell adhesion (Figure 1B) [25]. Interestingly, pulling Als molecules through their amyloid sequence, rather than through their terminal region, yielded force plateau signatures corresponding to the mechanical unzipping of amyloid β-sheet interactions involved in cell-cell adhesion (Figure 1C) [29].

AFM has also been used to stretch bacterial pili [30]–[34], cell surface appendages that play an important role in mediating the attachment of pathogens to host cells. Force spectroscopy experiments revealed that Gram-negative bacterial pili are highly extensible as a result of the unfolding of their helical quaternary structure [30]–[33]. This elongation may help the cells to redistribute external forces to multiple pili, thereby enabling them to withstand shear forces. By contrast, pulling on pili from the Gram-positive bacterium *Lactobacillus rhamnosus* GG yielded sharp adhesion force peaks with linear shapes, demonstrating that the pili do not unfold but instead behave as stiff nanosprings (Figure 1D) [34]. Presumably, these remarkable mechanical properties are used by the bacteria to resist physiological forces while being engaged in bacterial-host interactions. In summary, AFM has helped to shed new light on the adhesive and mechanical properties of cell surface adhesins engaged in pathogen-host interactions. These single-molecule analyses could pave the way in designing new molecules that block pathogen-host adhesion, which would offer a new means of preventing infection.

## Perspectives

The studies surveyed here demonstrate that AFM has great potential in microbial research. AFM imaging enables microbiologists to decipher the nanoscale architecture of cell surfaces and its remodelling upon growth or interaction with drugs. Moreover, force spectroscopy allows us to understand how cell surface receptors are spatially organized (e.g. clustering) and respond to force (e.g. single specific bonds, sequential unfolding, zipper-like adhesion, and spring-like properties). Knowing these molecular properties is critical to our understanding of cell surface functions.

Many challenges remain to be addressed. As we have already discussed, further developments in high-speed AFMs [20]–[22] will allow us to track dynamic molecular processes on individual pathogens, such as drug-induced remodelling or force-induced receptor clustering. In parallel, novel quantitative imaging modes [35] will make it possible to simultaneously image the structure and the biophysical properties (e.g. elasticity, adhesion) of pathogens at high speed (a few minutes per image) and high resolution (a few nanometers). Another challenging issue will be to further develop and apply non-invasive single-cell probe methods [36], in which the AFM tip is replaced by a single microbial cell, thereby enabling researchers to quantify the forces driving microbe-microbe, microbe-solid, and microbe-host interactions.
